# Striatal Plasticity in L-DOPA- and Graft-Induced Dyskinesia; The Common Link?

**DOI:** 10.3389/fncel.2016.00016

**Published:** 2016-02-08

**Authors:** Daniella Rylander Ottosson, Emma Lane

**Affiliations:** ^1^Developmental and Regenerative Neurobiology, Department of Experimental Medical Science, Lund UniversityLund, Sweden; ^2^School of Pharmacy and Pharmaceutical Sciences, Cardiff UniversityCardiff, UK

**Keywords:** GID, LID, LTP, LTD, DA, 5-HT, animal model, 6-OHDA

## Abstract

One of the major symptoms of the neurodegenerative condition Parkinson’s disease (PD) is a slowness or loss of voluntary movement, yet frustratingly therapeutic strategies designed to restore movement can result in the development of excessive abnormal movements known as dyskinesia. These dyskinesias commonly develop as a result of pharmacotherapy in the form of L-DOPA administration, but have also been identified following deep brain stimulation (DBS) and intrastriatal cell transplantation. In the case of L-DOPA these movements can be treatment limiting, and whilst they are not long lasting or troubling following DBS, recognition of their development had a near devastating effect on the field of cell transplantation for PD.Understanding the relationship between these therapeutic approaches and the development of dyskinesia may improve our ability to restore function without disabling side effects. Interestingly, despite the fact that dopaminergic cell transplantation repairs many of the changes induced by the disease process and through L-DOPA treatment, there appears to be a relationship between the two. In rodent models of the disease, the severity of dyskinesia induced by L-DOPA prior to the transplantation procedure correlated with post-transplantation, graft-induced dyskinesia. A review of clinical data also suggested that the worse preoperational dyskinesia causes worsened graft-induced dyskinesia (GID). Understanding how these aberrant behaviors come about has been of keen interest to open up these therapeutic options more widely and one major underlying theory is the effects of these approaches on the plasticity of synapses within the basal ganglia. This review uniquely brings together developments in understanding the role of striatal synaptic plasticity in both L-DOPA and GID to guide and stimulate further investigations on the important striatal plasticity.

## Introduction

Parkinson’s disease (PD) is a degenerative disease typically associated with aging. Although previously considered a pure motor disorder, it is now widely recognized that the disease presents not only with the classically described motor impairments such as tremor, akinesia, postural instability and rigidity, but in addition most patients also suffer from a range of non-motor symptoms that can include depression, constipation, dysasthesia and in the later stages for many patients, dementia (Juri et al., 2008). The pathophysiology of the disease is typified by the loss of pigmented nigrostriatal dopaminergic neurons and also by the presence of ubiquitin and a-synuclein positive protein inclusions termed Lewy bodies (Spillantini et al., 1997). However, as with the increased awareness of the diversity of symptoms, pathophysiology is also more widespread than the oversimplified perception of PD as being a dopaminergic disorder; Lewy bodies are spread throughout the peripheral and central nervous systems and degeneration can be seen in noradrenergic, serotonergic and cholinergic nuclei although generally not to the extent of that in the substantia nigra. Evidence suggests that the Lewy body pathology spreads up through the brain stem and into the basal ganglia and then affects cortical areas (Braak et al., 2003). This theory has been accepted more widely with the recognition that this could explain the pattern of non-motor symptomatology, some which appears prior to the development of the motor condition. We do note here though that there is some controversy over the exact implication of the presence of Lewy bodies in the disease, as some unusual forms of PD (such as Parkin mutations causing early onset PD; Takahashi et al., 1994) present without Lewy body pathology and in contrast incidental Lewy body disease is defined as the presence of Lewy bodies without clinical signs of PD.

The most widely used treatments for PD focus on enhancing the activity of the remaining dopamine by preventing its breakdown [catechol-o-methyl transferase (COMT) or monoamine oxidase B (MAO) inhibitors] or supplying exogenous dopaminergic stimulation (in the form of the dopamine precursor L-DOPA or the dopamine D_2_-like receptor agonists). These therapies are highly effective in early stage disease but as the disease develops their efficacy is limited and side effects, some of which can be treatment limiting. Dopamine agonists are associated with a significant risk of impulse control disorders which are expressed differently in individual patients but have led to compulsive gambling, shopping and sexual activity (Weintraub et al., 2010). Increasing the dose of L-DOPA in combination with other anti-parkinson’s drugs is the most likely course of treatment as the disease progresses, but this is associated with a concomitant increase in the risk of abnormal involuntary movements known as L-DOPA-induced dyskinesia (LID). As a result of these limitations on current drug therapy, there is not only a drive towards a cure for PD, but also improved therapeutic strategies in the meantime. One such possibility is the transplantation of dopaminergic neurons into the striatum in an attempt to replace the missing source of dopamine. Initially the focus has been on the use of primary fetal tissues, which has been trialed clinically across Europe and the US, to prove the principle that the approach has long lasting clinical efficacy. Latterly, recognizing the limitations of primary tissues, this has developed into a desire to use alternative sources of dopaminergic neurons (i.e., stem cell based sources of cells), but this has yet to reach clinical trials.

**Figure 1:**
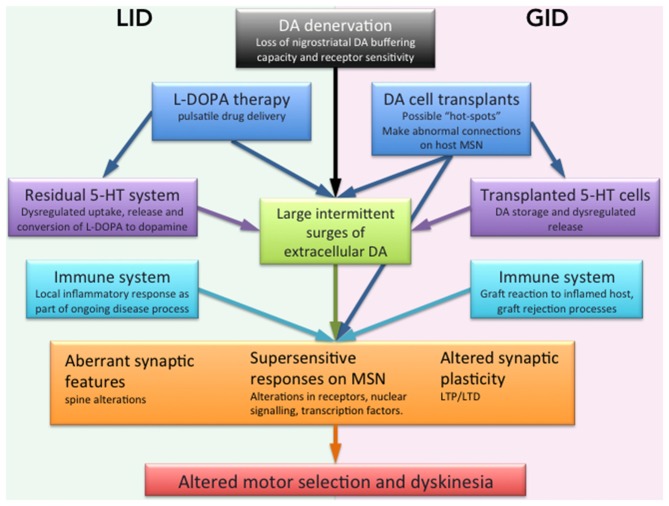
**Schematic illustration of the factors suggested to be involved in dyskinesia mediated by L-DOPA or dopaminergic cell transplantation.** A DA denervation such as that in PD causes the loss of DA buffering capacity by endogenous nigrostriatal terminals in the striatum. Together with pulsatile drug delivery from L-DOPA pharmacological administration. This leads to excessive swings of striatal DA, a causative factor for dyskinesia. Transplants may create inadequate or incomplete reinnervation from a graft or lack of synaptic integration may have a similar effect. The 5-HT system is proposed as having a role as a compensatory DA release site in the absence of residual DA axonal terminals in the striatum, similarly transplanted 5-HT neurons may also disrupt dopaminergic regulation. Our understanding of the inflammatory contributions are more limited but it is conceivable that low grade inflammatory responses present as an intrinsic part of the disease, or as a reaction to an incompatible element of the transplant contributes to aberrant neuronal activity. On the postsynaptic side of the striatal neurons (orange box) the stand supersensitive dopamine receptor responses as well as aberrant synaptic features on MSN spines and graft connections that altogether cause an altered synaptic plasticity with abnormal LTP and LTD which may underlie the abnormal behaviors seen in both LID and GID.

To date, in excess of 100 patients have received a fetal cell transplant, of those, many have been considered successful, (interpreted as allowing a reduction in/cessation of their anti-parkinson’s medication) and remaining better off for several years following the procedure (for review, see Barker et al., 2013). However, reports also emerged between 2001 and 2003 of the now-called “GID”, dyskinesia that were evident following transplantation *in the absence* of L-DOPA (Freed et al., 2001; Hagell et al., 2002; Ma et al., 2002; Olanow et al., 2003). The negative effect of this development on the cell transplantation field as a whole cannot be underestimated. However, this motor side effect exemplified by L-DOPA, and subsequently reinforced by cell transplantation, has further highlighted the need to understand the mechanisms underlying both induced disorders. Our understanding of dyskinesia in PD in both of these forms has developed considerably over the last 10 years and while the phenomenon of GID is relatively new, the intense scrutiny it has received has also shed light on mechanisms involved in its L-DOAP induced counterpart. One common feature of the two disorders may be the plasticity of striatal neurobiology in the dopamine-deprived environment of the putamen and the consequences of long term dopamine replacement. This review focuses on the role that striatal synaptic plasticity may play in the development of dyskinesia following both L-DOPA administration and cell transplantation (see Figure 1 for a summary of influential factors).

## Clinical Manifestation of Dyskinesia in Patients with Parkinson’S Disease

“Off medication” dyskinesia (defined as 12 h after the last drug administration), is a relative rarity in PD patients. In general, it presents mostly as early morning dystonia in around 16% of patients with a very small (1%) exhibiting choreic dyskinesia, which is unrelated directly to their medication regime (Cubo et al., 2001), but can be a complication of long term drug therapy. The more commonly associated dyskinesia in PD patients is the LID present during the period of L-DOPA activity during the day. Two main types of LID are described, the first, and best characterized being “peak dose dyskinesia”; monophasic, typically choreiform in nature, purposeless and non-rhythmic and often affecting the more severely parkinsonian side of the body to a greater extent. They appear as the plasma L-DOPA levels peak and are sustained until they decrease, typically covering much of the effective period of the drug (2–3 h). It is noteworthy though, that there is no direct correlation between the severity of peak dose dyskinesia and the exact peak plasma levels of L-DOPA reached, severity in fact being related to other factors including emotional stress (Mones, 1970; Nutt, 1990). The movements can be very intrusive and patients are highly averse to this in the early stages of their disease course making them very wary of L-DOPA, although this attitude does shift as dyskinesia become established and patients are faced with the choice of limited movement vs. mild dyskinesia (Khlebtovsky et al., 2012). The second form of LID is diphasic dyskinesia, appearing as the effective dose of L-DOPA onsets and wanes with a dyskinesia free period of symptomatic relief in between. This suggests, that in contrast to the peak dose dyskinesia associated with high plasma L-DOPA, these are associated with a critical but low level of circulating L-DOPA or dopamine, likely to be stimulating the increasingly sensitized dopamine receptors. Phenomenologically this form of dyskinesia are more typically associated with the lower limbs, and are more dystonic in nature, often affecting patients with a younger onset of disease. There is a lot less known about diphasic LID and hereon in LID will be used to refer to peak dose LID.

The exact incidence of peak dose LID in PD is hard to estimate, given the variance in trial design and lack of recognition of mild dyskinesia by patients themselves (Pietracupa et al., 2013). Indeed estimates vary from 0% up to 50% of patients in the first year of treatment (reviewed in Manson et al., 2012), to 94% at 15 years of treatment (Hely et al., 2005), while others estimate the risk to be around 10% per year of L-DOPA exposure (Ahlskog and Muenter, 2001). In those patients that are affected, the impact of LID on quality of life is clear from several studies, affecting mobility, communication and emotional well-being (Encarnacion and Hauser, 2008; Montel et al., 2009). There is no doubt that it is a hugely variable condition and it is still accepted that LID, in some form, are an almost inevitable part of PD for most patients. The only available treatment is the anti-viral amantadine, which has some benefit for a period of time but long-term efficacy is limited. Nevertheless, with a greater understanding of the medications and tools at the disposal of clinicians, patients with overt, severe dyskinesia are a relative rarity compared to years gone by. The remaining concern is that patients may not be receiving the full potential anti-parkinsonian benefit of the drugs on offer, as treatment is limited in order to effectively control the risk of LID. Importantly, as new therapies are developed the plastic changes that long term L-DOPA evokes in the brain may influence the efficacy of these therapies or the side effects they generate.

As a result of the limitations LID generation imposes on L-DOPA treatment, and to ameliorate some of the symptoms that L-DOPA does not address (tremor being the classic example), alternative therapies are constantly being explored. One significant surgical intervention, which has become used world-wide over the last 15 years, is deep brain stimulation (DBS) in which electrodes delivering high frequency stimulation are implanted bilaterally into the subthalamic nucleus. This has the immediate effect of reducing motor symptoms and alleviating the need for high dose L-DOPA therapy and many patients are able to reduce their medication significantly once the stimulators are turned on, and consequently their LID. There have been case reports of both DBS and subthalamotomy in which L-DOPA independent dyskinesia were observed following surgery, although resistant to amantadine these movements resolved themselves, in one case within minutes of turning off the stimulator (Brodsky et al., 2006), in others, within 6 months of the surgical intervention (Merello et al., 2006). These data are useful in that they give clues as to how the pathophysiology may vary from that of LID, which is amantadine responsive to a degree. DBS is not a focus for the review but worthy of mention as it demonstrates clearly that alternative basal ganglia circuitry may contribute to the generation of dyskinesia.

A third and further cause of dyskinesia in PD has been described over the last 14 years, an intractable form of dyskinesia that has appeared as a result of intrastriatal fetal cell transplantation. The first published report in 2001 reported “runaway dyskinesia” 6–12 months post transplantation in five patients (out of a total transplanted cohort of 33; Freed et al., 2001). Persistent generalized dyskinesia or movements specifically located to the arm was described. This was followed up by two other clinical trials reporting hyperkinetic movements and repetitive stereotypic movements of the limbs (Hagell et al., 2002; Olanow et al., 2003), one follow up report likened the movements to those observed during biphasic dyskinesia, and similarly inferred that the cause might be suboptimal levels of dopamine (Olanow et al., 2009), as opposed to the initial hypotheses of excessive or patchy putaminal dopaminergic innervation (Ma et al., 2002).

For the majority of patients, the overall severity of these movements was clinically considered to be relatively mild, especially in comparison to L-DOPA induced dyskinesia. Many patients failed to recognize the symptoms themselves, and in a European cohort the full incidence required independent review of video footage (Hagell et al., 2002). In the majority of cases the patients’ responses to amantadine are not well documented, but only a handful have gone on to require additional interventions, which have been DBS of either the STN or GPi. Although the incidence may appear relatively small, this is out of a reported total of less than 70 patients, giving an average frequency of 39% across three clinical trials (more have been transplanted worldwide but with limited follow up and lack of consistent and complete reporting; Lane et al., 2010). Taking into account some implicit reports of aberrant movements post transplantation (reviewed in Lane et al., 2010) this average across six trials is still 32% of patients and therefore demonstrates a significant concern in the development of transplantation as a reliable and safe therapy for PD.

## Animal Models of Dyskinesia

Before going on to discuss current knowledge of the mechanisms underlying LID and GID, it is worth highlighting how these types of dyskinesia are represented in the most commonly used animal models of PD (reviewed in more detail in Carta et al., 2006; Smith et al., 2011 and elsewhere). Historically both the 6-OHDA lesioned rodent and MPTP-treated primate have been used as models for peak dose LID, whilst the study of surgically related dyskinesia (DBS and transplantation) has, for practical reasons, been studied more selectively in the rodent model. Importantly there are limited studies of the mechanisms underlying end of dose or diphasic LID, and they are not well represented in animal models. The longevity of the peak dose LID model is supported by excellent face validity in the primate in particular (Carta et al., 2006) but also clear behavioral and neurochemical parallels can be identified in the rodent models (Cenci et al., 1998). Although in many cases neurobiological changes identified in the models are also observed in patient materials, the resulting development of effective novel therapeutic approaches has been frustratingly elusive, with many therapies failing at early clinical stages (Fox et al., 2006). At least in models of LID there is a clinically limited but effective pharmacological therapy in the form of amantadine, against which the animal models can be, and have been validated (Blanchet et al., 1998; Lundblad et al., 2002), the same cannot be said for GID for which no such validation is possible. Developing an animal model of GID is more complex. In patients the behavior is spontaneous and presents in the absence of drugs, yet observational studies in both primates and rodents with dopaminergic lesions and fetal cell transplants have not revealed any consistent, persistent abnormal behaviors (Lane et al., 2006; Redmond et al., 2008; Vinuela et al., 2008). Cost and practical limitations have thwarted attempts to explore GID in detail in primates, while two models of GID exist in rodents but both of which depend upon the exogenous administration of drugs to elicit the abnormal responses directly contrasting the “off medication” status of patients. The administration of L-DOPA and amphetamine post-transplantation produces different behaviors in the rat following transplantation that have been used as a proxy measure for true GID (Lane et al., 2006; Maries et al., 2006). This is discussed in more detail elsewhere (Lane, 2011 and others) but highlights the complexity of preclinical investigations into this field. In as far as is practically possible, some patients with GID have been reviewed and there has been a very interactive process to take hypotheses based on this patient data into preclinical studies and* vice versa*. There is however the significant lack of post-mortem study of any patient with cell transplantation and a recorded development of GID.

## The Striatum, a Place of Heterogenous Plasticity

The caudate putamen, a single anatomical entity in rodents, is composed of a complex mosaic organization that can be divided into several functional compartments in rodents based on (i) different cortical afferents e.g., to the ventromedial vs. the dorsolateral striatal subregion; (ii) the patch and matrix compartments; or (iii) the direct or indirect efferents of the striatal medium-sized spiny neurons (MSNs; Gerfen, 1992; Crittenden and Graybiel, 2011), all of which must be considered for their role and vulnerability in the face of long term L-DOPA administration. There are indications that the striatal plasticity that underlies dyskinesia induced with L-DOPA or graft share the same neuropathologies or at least are linked with each other. Firstly, most transplantation patients experiencing GID had presented preoperatively with established LID; secondly, prior exposure to L-DOPA and development of LID is necessary for, and correlated to, the induction and severity of GID post transplantation in rodent models (Lane et al., 2009; Steece-Collier et al., 2009; García et al., 2011a). Potentially mechanistically key to this relationship between LID and GID, are the plastic changes which occur in subregions of the striatum in response to repeated, pulsatile exposure to L-DOPA administration and that correlate with the development of LID.

Dyskinesia can be seen as an aberrant form of plasticity where DA derived from L-DOPA or possibly transplanted neurons, causes long-lasting molecular and behavioral changes in the dopamine denervated environment (see Figure 1). This is particular evident on glutamatergic synapses where both pre and postsynaptic mechanisms of the MSN are involved. Accordingly, dyskinesia is improved by treatments that either stabilize extracellular DA levels or dampen the supersensitive response of MSN. The relative contribution of pre- vs. postsynaptic mechanisms may differ among dyskinetic subjects, making them more or less responsive to different treatment options. Presynaptically, a lack of autoregulatory machinery for the DA release leads to excessive swings of extracellular DA after L-DOPA administration that has been considered a primary cause of dyskinesia (Lindgren et al., 2010). In the severely DA denervated brain L-DOPA can even instead be taken up, converted and released by 5-HT axonal terminals that compensate for the denervation of their neighbor DA neurons (see “Discussion” below; Figure 1). The postsynaptic plasticity includes upregulation of transcription factors and plasticity genes in the striatal MSNs with changes to striatal D1 and D2 dopamine receptor expression (Blanchet et al., 1995; Andersson et al., 1999; Konradi et al., 2004; Cenci and Lundblad, 2006). This increased sensitivity is reflected in abnormally high and sustained levels of both opioid precursor genes and ΔFosB-like transcription factors (Doucet et al., 1996; Andersson et al., 1999; Hagell and Cenci, 2005; Lindgren et al., 2011). Once instigated, these alterations in receptors levels remain, even in the absence of dopaminergic stimulation. Behaviorally this is reflected clinically in a rapid reinstatement of LID upon re-exposure to L-DOPA after a period of withdrawal in animal model (Andersson et al., 2003). Similarly in the clinic a drug holidays from L-DOPA fail to improve the incidence of LID (Rascol, 2000). The upregulation of receptor signaling is accompanied by a pronounced activation of downstream elements to the D1 receptor, e.g., ERK1/2 (Pavón et al., 2006; Santini et al., 2007; Westin et al., 2007). D1 receptor signaling pathway has recently shown to be modulated by the D3 receptor, that thereby could modulate the development of LID (Solís et al., 2015). This D2 subclass family member has low levels in dorsal striatum but there has been evidence of increase receptor expression after L-DOPA dyskinesiogenic treatment (Bezard et al., 2003).

Even if the striatum is generally considered to be a homogenous structure recent developments have shown an important heterogeneity that should be taken into account when studying striatal plasticity and also synaptic plasticity (see below). Initial analyses of anatomical and biochemical data pointed to the lateral striatum has having a specific role in the generation of these abnormal behaviors. Upregulation of Na^+^K^+^-ATPase occurs specifically in the lateral striatum suggesting increased neuronal activity of MSNs in this location (Konradi et al., 2004). An increased expression of glutamate transporter that could underlie an increased glutamate release, also implicates this region. Similarly a striatal induction of ΔFosB affects primarily the lateral striatum and the direct pathway MSNs in dyskinetic animals (Doucet et al., 1996; Andersson et al., 1999), consistent with the upregulation of D1 receptors. These and other data imply that LID may result from high, intermittent stimulation of striatal dopamine receptors causing sustained downstream changes in gene and protein expression (Cenci et al., 1998; Konradi et al., 2004). While the degree of nigrostriatal dopaminergic loss is a strong causative factor in LID, strength for a post-synaptic root cause for LID also comes from other conditions in which LID have been reported. L-DOPA is also used in the management of inherited defects of dopamine synthesis and DOPA-responsive dystonia and LID have been reported in both conditions (Hwang et al., 2001; Pons et al., 2013). The LID are phenomenological similar in their presentation but importantly the dopaminergic terminals are still intact and presumably able to regulate the conversion of L-DOPA to dopamine and the release of dopamine into the synaptic cleft. Although there is much to learn about these conditions it would implicate the post synaptic receptors and abnormal stimulation as a potential cause (Bezard, 2013).

More detailed regional analysis has subdivided the lateral striatum into ventro and dorso-lateral areas and highlighted specific LID-related plasticities in the ventrolateral areas. Among these have FosB expression selectively in ventrolateral areas been linked to forelimb and facial LID (Maries et al., 2006). Moreover, alterations in the glutamate receptors on the postsynaptic compartments with increased expression of N-methyl-D-aspartate (NMDA) and AMPA 2-amino-3-(5-methyl-3-oxo-1,2-oxazol-4-yl)propanoic acid subunits in the ventrolateral compared to the dorsolateral striatum (Rylander, 2013). These differences in subunit expression turned out to have a particular role in the later discussed corticostriatal synaptic plasticity (Calabresi et al., 2007; Cenci and Konradi, 2010) shifting the probability and degree of LTP in striatal compartments (Rylander, 2013). These data demonstrate that striatum is not as a homogenous region as previously believed and region specific changes could easily be missed.

It has been hypothesized that the plastic changes initially induced with LID can also be linked to the generation of GID. In this hypothesis inhomogenous dopamine re-innervation of the host-putamen after dopamine fetal transplantation are believed to cause excessive dopamine release in “hotspots” that in turn would activate the aforementioned supersensitive signal transduction mechanisms of the dopamine-signal pathway similar to LID (Ma et al., 2002). This is based on early findings that prior long-term exposure to L-DOPA may already have induced this altered profile of dyskinetic behavior prior to transplantation. In support of this, amphetamine-induced rotational behaviors following transplantation (as a model for GID in rodents), are associated with an abnormally high expression of c-Fos in striatal regions innervated by the graft-derived DA axons (Cenci et al., 1992; Muñoz et al., 2003; Lane et al., 2009). However, if this were the only explanation for GID we might expect to see an irregular downregulation of the supersensitive dopamine receptors relating areas of high and low reinnervation, or a correlation between c-Fos expression and the expression of non rotational abnormal movements. Certainly in rodents this has not been the case, there is no correlation between the c-Fos activation and generation of amphetamine-induced abnormal movements and both fosB and dopamine receptor levels appear to normalize relatively evenly in the areas of graft innervation (Lane et al., 2009; Smith et al., 2012). Even good grafts are currently not able to innervate with complete homogeneity throughout the striatum so it remains possible that non-reinnervated areas remain supersensitive and trigger these behavioral response. Although the relationship of GID to pre-existing LID is reasonably strong, thus far the mechanisms are elusive, and it may lie in other areas of plasticity.

## Synaptic Plasticity in LID

The corticostriatal synapse between the glutamatergic cortical efferent and striatal MSNs has proven to be essential for movement improvement after L-DOPA or transplantation of dopaminergic cells. In animal models has it been possible to examine the functional and morphological state of these synapses and using these has shown severe morphological alterations of the striatal MSNs, including shrinkage and loss of dendritic small protrusions, the spines in the PD state (Ingham et al., 1998; Fieblinger et al., 2014) . The intrinsic excitability and synaptic connectivity has further been altered in the PD state and in LID (Fieblinger et al., 2014). Structural changes cause loss or reorganizations of the corticostriatal synapses and thereby interfere with its signaling through the classical bidirectional synaptic plasticity, i.e., long-term potentiation (LTP) and long-term depression (LTD). Both LTP and LTD are widely expressed at excitatory synapses throughout the brain and have been described at corticostriatal synapses where they are believed to underlie motor-skilled learning, cognitive performance and reward mechanisms (Calabresi et al., 2007). The induction of synaptic plasticity here requires interaction of DA and other neurotransmitters including glutamate function through ionotrophic (NMDA and AMPA) as well as metabotropic receptors but also acetylcholine, nitric oxide and endogenous cannabinoids (Calabresi et al., 2007; Shen et al., 2015). Not surprisingly are both LTP and LTD absent or severely altered in rats with a dopaminergic denervation (Picconi et al., 2003; Paillé et al., 2010) but whether this loss of synaptic plasticity can be fully restored by DA pharmacotherapy or cell therapy has not been extensively studied: in rats with 6-OHDA lesion a restoration in LTP has been seen in the dorsolateral striatum with chronic L-DOPA administration, a situation which is then confounded by the development of LID (Picconi et al., 2003). Certainly, the initial loss of spine and dendrites on the MSNs after a DA-denervation may impede a proper restoration of the synaptic connections by any dopaminergic therapy, L-DOPA or DA fetal transplants (Zhang et al., 2013) as this is the main site of dopamine/glutamate interaction.

As one of the consequences to the morphologically altered MSNs a non-physiological corticostriatal plasticity has been repeatedly shown in both rodent models and in patient with LID (Calabresi et al., 2007; Morgante et al., 2006). In the rat PD model, LID associates to irreversible LTP of the striatal MSN, which fails to depotentiate (Picconi et al., 2003) and with persistent deficits in LTD in the same neurons (Picconi et al., 2011). A recent paper further links an aberrant LTP in specifically the direct pathway MSN with LID and involve a cholinergic muscarinic receptor modulation (Shen et al., 2015). Also, in the clinics, has deficit in LTP-like plasticity been detected but then in the motor cortex. Only after L-DOPA treatment that did not cause dyskinesia could the plasticity be normalized (Morgante et al., 2006).

In an attempt to unravel the mechanisms behind LID associated plasticity, one has looked into alterations in the glutamate receptors that are involved in LTP and LTD. Here, an increased expression of a particular AMPA receptor subunit, i.e., the alternatively spliced calcium-permeable subunit, has been linked to LID in the rat (Kobylecki et al., 2013). Also, striatal NMDA/AMPA receptor ratio and abnormal receptor distribution between synaptic and extrasynaptic sites might disturb the function of the glutamatergic synapse and account for the pathological plasticity seen in LID (Gardoni et al., 2006; Mellone et al., 2015). L-DOPA treatment fails to rebalance the physiological synaptic parameters in NMDA/AMPA receptor ratio after a PD lesion, in contrast to a non-dyskinesiogenic treatment (Bagetta et al., 2012). Adding further weight to the relationship between this impaired signaling and the behavioral output correcting the NMDA subcellular distribution through the use of cell permeable peptides has been associated with attenuation of LID (Gardoni et al., 2012). Recent developments have pointed to specific structural changes of dendritic spines in the striatum suggesting a disrupted balance between direct and indirect pathway neurons and an aberrant action selection in the basal ganglia. Here, using combined electrophysiology and three-dimensional reconstructions of transgene labeled MSNs, studies have pointed to a remarkable regrowth of spines on the indirect pathway neurons after L-DOPA treatment that was initially lost by the DA denervation, a regrowth that is correlated to dyskinesia in mice (Suárez et al., 2014). In contrast, the direct pathway neurons show no change in spine numbers but instead an elevated excitability in PD and supressed excitability in LID models (Fieblinger et al., 2014; Suárez et al., 2014). Data by Zhang et al. (2013) further links dyskinesia to altered synaptic contacts of the corticostriatal glutamatergic synapses onto the spines of MSNs. An imbalance between corticostriatal and thalamostriatal pathways is here proposed to contribute to the persistent LTP seen in dyskinesia. Ultimately it is clear that the structure and receptor composition on the corticostriatal synapses are an important component recovery from dyskinesia.

## Synaptic Plasticity After Cell Transplantation in PD

Even though transplanted neurons are able to innervate the host striatum, release DA, and reverse alterations in neuropeptide expression after a dopamine denervating lesion (Cenci et al., 1993) it is unclear whether they restore the synaptic network in the PD brain or if they cause further alterations in synaptic plasticity. The importance of this question lies in the fact that a functional integration from the grafted neurons into the host microcircuits with formation of bidirectional synaptic contacts between the host and grafted neurons would give superior therapeutic benefit than a mere neurochemical restoration (Rylander et al., 2013). Furthermore it will be of great relevance for novel cell therapies that are currently developing e.g., human embryonic stem cell derived neurons that have been shown to generate DA neurons with an authentic midbrain phenotype that survive transplantation and can restore motor deficits and synaptically be integrated from host neurons (Kriks et al., 2011; Grealish et al., 2014). It has been suggested that neural grafts go beyond that of simple DA delivery and involves more complex mechanisms of functional integration that lead to more substantial motor recovery. A clinical study has shown a delayed recovery in motor cortical activity occurring first after 18 month post-transplantation compared with the more direct increase in DA storage capacity that was detected with ^[18]^F-dopa position emission tomography by 6 months post-transplantation (Piccini et al., 2000). Also, in animal models of PD, the capacity for DA synthesis and storage in the grafts is detected well before a significant improvement in complex sensory motor behavior (Annett et al., 1995; Winkler et al., 1999).

**Figure 2:**
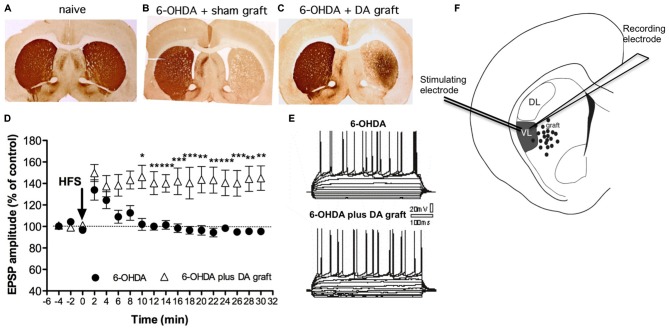
**Synaptic plasticity can be restored by fetal transplants.** Adapted from Rylander et al. (2013), with permissions. **(A–C)** Photos from TH immunohistochemistry show the growth of transplanted DA neuron projections in striatum. The 6-OHDA lesion produced a unilateral DA denervation **(B)** that DA graft were able to partly reinnervate **(C)** along with a gradual improvement in akinesia (not shown). **(D–F)** Grafted DA neurons can restore LTP close to the graft. In the reinnervated region electrophysiological recordings of striatal medium-sized spiny neurons (MSN) show a restoration in long-term potentiation (LTP) in DA grafted animals (triangles) compared to animals with 6-OHDA lesion only. Left panel represents the traces for the evoked excitatory post-synaptic potential (EPSP) pre- and post HFS (the stimulating protocol for LTP induction).

Few studies have explored cell-transplant integration in PD brain using electrophysiology. Nevertheless’ recent advances in optogenetic and chemogenetic tools as well as transynaptic labeling (Tønnesen et al., 2011; Grealish et al., 2014; Torper et al., 2015) combined with electrophysiology allows for more delicate measurements of transplants integration in the future. Previous extracellular electrophysiological recordings have shown that dopaminergic fetal neurons partially restore spontaneous neuronal firing in the striatum up to 2 mm from the core of the grafted area (Di Loreto et al., 1996; Stromberg et al., 2000) and also restore neuronal activity in the subthalamic nucleus (Rumpel et al., 2013). Yet only one study has explored the synaptic plasticity in striatal MSNs after fetal cell transplantation (Rylander et al., 2013). Here transplants of DA neurons were shown to partially restore activity-dependent LTP (see Figure 2D) in contrast to 5-HT-rich transplants. Importantly to the field of PD cell-therapy, the restoration of LTP was accompanied with a partial restoration in motor deficits in rats at 7–10 weeks post-transplantation (Figure 2). The restoration in synaptic plasticity was specifically seen in the ventrolateral striatum that received sufficient DA fiber innervation from the transplants and where grafted fibers are known to form synapses (Doucet et al., 1989). In the dorsolateral striatum, where the DA re-innervation was less (about 45%), the grafted DA neurons were not able to restore any type of synaptic plasticity deficits. Taken together these data suggest that the function of DA transplants goes beyond that of simple DA delivery and involves more complex mechanisms of functional integration that lead to more substantial clinical recovery. This most probably involve restoration of synaptic plasticity and possibly forming anatomically appropriate connections capable of influencing host behavior. Yet is still not known whether or not restoration in synaptic plasticity occurs as a consequence of altered synaptic rearrangements or as a precise reconstruction of the original physiological connections or how that would be affected by dyskinesia as these rats were not tested for that.

Based on the results above, the restoration of synaptic plasticity could be seen as an indicator for successful graft integration that underlies motor improvement. The fact that the restoration was limited to the most richly reinnervated regions might explain the limited efficacy of DA transplants in alleviating clinical symptoms and supports a multisite-grafting procedure to more extensively restore the plasticity in the whole parkinsonian putamen (Rylander et al., 2013). Indeed multisite transplantation of DA graft is superior to single-site “hot-spot” DA grafts because it lead to functional recovery and lower incidence of GID (Maries et al., 2006). This notion is supported by a clinical PET study (Ma et al., 2002). On the other hand the incidence of GID is positively correlated with the amount of grafted tissue (Hagell et al., 2002; Lane et al., 2006). Hence there seem to be a delicate balance between functional integration of the grafted neurons with motor restoration on one hand and development of GID with possible pathological synaptic integration on the other.

Previous studies have evidenced that striatal DA grafts form aberrant connections with host MSNs (Freund et al., 1985; Mahalik et al., 1985) that has further been correlated with GID (Soderstrom et al., 2008). Transplanted DA neurons synapse onto MSNs with increased proportion of asymmetrical and perforated synapses in both parkinsonian rats and monkeys (Leranth et al., 1998; Soderstrom et al., 2008), synaptic specializations that are exacerbated in rats with GID-like behaviors (Soderstrom et al., 2008). Asymmetrical synapses in turn have been associated with excitatory neurotransmission (Peters and Palay, 1991) and perforated synapses with elevated excitatory activity and LTP (Marrone and Petit, 2002) raising the possibility that the synaptic connection would alter the synaptic plasticity within the grafted region to a form that is associated with dyskinesia (Picconi et al., 2003; Morgante et al., 2006). As the morphological aberrations occurs via a dysregulation of Cav1.3 L-type calcium channel, the aberration can be prevented by the *in vivo* administration of an L-type calcium channel antagonist (Soderstrom et al., 2010). This reduces not only LID (Schuster et al., 2009) but also the development of GID in the rat (Soderstrom et al., 2010). The data suggests that improved synaptic connection between the transplants and host neurons would effectively relieve PD symptoms through restoration of synaptic plasticity.

## Serotonin and Dyskinesia

There are several non-dopaminergic systems that have shown to play important roles in the development of dyskinesia, among which a major player is the serotonin (5-HT) system. There is both a variable loss of 5-HT in the parkinsonian brain and significant numbers of 5-HT neurons are transplanted alongside the dopaminergic neurons in the primary graft preparation, creating a dynamic of host striatal serotonin deficiency and excessive post transplantation innervation that has to be reconciled. The 5-HT system has a remarkable capacity for compensatory sprouting after either a serotonergic or dopaminergic denervation (Radja et al., 1993; Maeda et al., 2005) and recently a maladaptive plasticity of this system has been linked to LID (Rylander et al., 2010). Long-term L-DOPA pharmacotherapy to the PD brain causes an increased striatal density of 5-HT axonal fibers that has been positively correlated with the severity of LID in both rat and primate models as well as in post-mortem tissue from PD patients (Rylander et al., 2010). The results have been supported by a recent clinical study where increased binding of [^11^C]DASB PET binding indicating a higher 5-HT fiber density was detected in patients with LID compared to stable L-DOPA responders (Politis et al., 2014).

Recent discoeries from rat and primate models of PD suggest multiple mechanism of action through which the 5-HT system may contribute to LID. Of these the DA-releasing role has emerged as one of the concepts. In the DA-denervated brain the serotonergic terminals are especially good candidates for converting exogenously administered L-DOPA into DA (Tanaka et al., 1999; Lindgren et al., 2010). The release from the 5-HT terminals facilitates the therapeutic action of L-DOPA by facilitating the conversion of L-DOPA to DA and thus relieving PD motor symptoms, but the raphe-striatal serotonergic terminals lack an auto-regulatory feedback mechanism for DA release and cause an aberrant release with excessively high DA levels in the extracellular space as a consequence. Such fluctuations, between high and low DA levels may compound the pulsatility of dopamine in the brain and exacerbate receptor sensitivity and therefore the responses of striatal neurons to trigger LID (Carta et al., 2007; Lindgren et al., 2010).

The developing dorsal raphe is close in proximity to the ventral mesencephalic area dissected and suspended for transplantation in PD and are hard to exclude in their entirety during the dissection without compromising the yield of dopaminergic neurons. Post mortem and imaging data illustrates that these 5-HT neuronal populations vary in extent between transplanted cohorts but can be extensive (Mendez et al., 2005; Politis et al., 2010). As 5-HT grafted neurons do not form synaptic contacts to the same extent than DA-grafted neurons (Mahalik and Clayton, 1991) but still retain the capability to release excessive amounts of unregulated DA it has been postulated that they may contribute to the development of post-transplantation dyskinesia. Importantly, preclinical data shows no correlation between grafted 5-HT content and GID (Lane et al., 2006) and a negative effect is only seen with an unfavorable DA/5-HT ratio (Carlsson et al., 2007; García et al., 2011b). Accordingly, clinical PET imaging studies have reported prominent graft-derived 5-HT innervations and a higher 5-HT/DA ratios in patients with GID (Politis et al., 2010; Politis and Loane, 2011; although it must be noted that no patients with successful grafts without GID have been scanned for comparison). This story is less than complete, as some data indicates that grafts with some 5-HT content produce better functional recovery and pharmacological assays to try and tease out this relationship have been inconclusive. Low doses of the 5-HT_1A_ autoreceptor agonist buspirone were able to attenuate the GID observed in patients (Politis and Loane, 2011) but preclinical studies suggest that this may occur through a dampening aberrant activity of the dopamine receptors. The 5-HT reuptake inhibitors fluvoxamine significantly increases the severity of GID (Lane et al., 2006) and direct activation of 5-HT_1A_ and_1B_ auto-receptors significantly reduces GID in rats via the presynaptic host-derived 5-HT system (Shin et al., 2012). However, while transplanted rats show an attenuation of amphetamine-induced abnormal movements upon buspirone administration in the same way as patients GID is reduced, this effect seems to be independent of the 5-HT system and rather acting via the DA D_2_ receptor (to which buspirone acts as an antagonist; Shin et al., 2012). These results point to a critical role of both 5-HT1 and D2 receptors in the modulation of GID, and suggest that 5-HT neurons may exert a modulatory role (Shin et al., 2012).

## Inflammation and Dyskinesia

Inflammatory processes are certainly active in the parkinsonian brain with pathological evidence of upregulated microglia and positive benefit from long term administration of non-steroidal anti-inflammatories (reviewed in Wang et al., 2015). However, there is limited understanding of the role of the immune system in the generation of LID likely to be due to the lack of an appropriate animal model. The only clinically available treatment for LID, amantadine has been shown to have anti-inflammatory properties (Ossola et al., 2011) and other approaches have found that molecules with anti-inflammatory activity may help reduce LID severity or onset (Barnum et al., 2008; Aron Badin et al., 2013; Bortolanza et al., 2015). Importantly in the context of striatal plasticity anti-inflammatories have been shown to improve spine abnormalities in mouse models of Huntington’s disease, a link that may be worth further investigation (Simmons et al., 2013).

Clinical observations have coupled GID to the host immune system; GID has been reported in patients that received no immunosuppression (Freed et al., 2001) or soon after withdrawal of immunosuppression, suggesting that some degree of graft rejection and local inflammatory response may have caused GID (Olanow et al., 2003; Hagell and Cenci, 2005; Piccini et al., 2005). In animal models, Soderstrom et al. (2008) revealed novel dyskinetic behaviors, i.e., orolingual and contralateral forelimb dyskinesia that emerged and increased with escalating immune activation using allografts with peripheral spleen cell injections, whereas the inflammatory response itself using proinflammatory cytokines or orthotopic skin allograft is not sufficient to evoke dyskinetic behaviors (Lane et al., 2008).

Ultrastructural analysis using electron microscopy shows a positive correlation between GID severity and proportion of aberrant synaptic features in the parkinsonian striatum. Instead of synapsing onto the neck of the MSNs spines (and glutamatergic endings to the head) grafted DA neurons formed new synapses onto the dendrites directly instead of spines. This change was found enhanced with immune activation and to be significantly correlated with GID (Soderstrom et al., 2008). Inflammation may thus interfere with the appropriate synapse formation between graft and host-neurons. Indeed, elevated expression of synaptogenesis genes is linked to inflammation and inflammatory factors can increase the synaptic strength and AMPA receptor trafficking (Beattie et al., 2002; Yukhananov and Kissin, 2008). Aberrant synaptic profiles that might excessively excite the activity within the grafted striatum can give rise to aberrant focal output similar to the sustained LTP seen in LID (Picconi et al., 2003). A consideration for future clinical trials should therefore be a careful evaluation of the value of long-term immunosuppression following neural transplantation or perhaps attempting to prevent the dendritic spine loss using calcium channel antagonists.

## Conclusion

L-DOPA causes long term changes in the anatomical and neurochemical make up of the striatum and in particular corticostriatal connections, all of which have been correlated to LID. Although complex, there has been some work trying to identify how these changes may influence the development of GID. Critically, avoiding patients with established significant LID may be the current pathway to successful transplantation; a model being adopted by the ongoing EU funded clinical trial TransEuro. One of the major limitations in understanding both LID and in particular GID has been the animal models and the parity between them and the patients. Currently our models of PD are oversimplified and nigrostriatal denervation alone is unlikely to be providing us with the true physiological insight into a patients’ brain. Nevertheless, exploring both LID and GID is giving us a greater understanding of exactly how the brain is capable of changing in certain environments and the functional consequences that result. Whether we can undo these changes, or only work to avoid their occurrence in the first place, remains to be seen.

## Author Contributions

EL wrote and collected data for clinical manifestation of GID and LID and explained the animal model. DRO wrote and collected data for striatal plasticity and synaptic plasticity in dyskinesia and the serotonin system. EL and DRO together made the figures and wrote the inflammation part.

## Funding

EL has no financial disclosure. DRO is supported by Swedish Society for Medical Research for postdoctoral fellowship.

## Conflict of Interest Statement

The authors declare that the research was conducted in the absence of any commercial or financial relationships that could be construed as a potential conflict of interest.

## References

[B1000] Ahlskog J. E. Muenter M. D. (2001). Frequency of levodopa-related dyskinesias and motor fluctuations as estimated from the cumulative literature. Mov. Disord. 16, 448–458. 10.1002/mds.1090 11391738

[B1] Andersson M. Hilbertson A. Cenci M. A. (1999). Striatal fosB expression is causally linked with l-DOPA-induced abnormal involuntary movements and the associated upregulation of striatal prodynorphin mRNA in a rat model of Parkinson’s disease. Neurobiol. Dis. 6, 461–474. 10.1006/nbdi.1999.0259 10600402

[B2] Andersson M. Westin J. E. Cenci M. A. (2003). Time course of striatal ΔFosB-like immunoreactivity and prodynorphin mRNA levels after discontinuation of chronic dopaminomimetic treatment. Eur. J. Neurosci. 17, 661–666. 10.1046/j.1460-9568.2003.02469.x 12581184

[B3] Annett L. E. Torres E. M. Ridley R. M. Baker H. F. Dunnett S. B. (1995). A comparison of the behavioural effects of embryonic nigral grafts in the caudate nucleus and in the putamen of marmosets with unilateral 6-OHDA lesions. Exp. Brain Res. 103, 355–371. 10.1007/bf00241495 7789442

[B4] Aron Badin R. Spinnewyn B. Gaillard M. C. Jan C. Malgorn C. Van Camp N.. (2013). IRC-082451, a novel multitargeting molecule, reduces L-DOPA-induced dyskinesias in MPTP parkinsonian primates. PLoS One 8:e52680. 10.1371/journal.pone.0052680 23300984 PMC3536787

[B5] Bagetta V. Sgobio C. Pendolino V. Del Papa G. Tozzi A. Ghiglieri V.. (2012). Rebalance of striatal NMDA/AMPA receptor ratio underlies the reduced emergence of dyskinesia during D2-like dopamine agonist treatment in experimental Parkinson’s disease. J. Neurosci. 32, 17921–17931. 10.1523/JNEUROSCI.2664-12.2012 23223310 PMC6621675

[B6] Barker R. A. Barrett J. Mason S. L. Björklund A. (2013). Fetal dopaminergic transplantation trials and the future of neural grafting in Parkinson’s disease. Lancet Neurol. 12, 84–91. 10.1016/s1474-4422(12)70295-8 23237903

[B7] Barnum C. J. Eskow K. L. Dupre K. Blandino P. Jr. Deak T. Bishop C. (2008). Exogenous corticosterone reduces L-DOPA-induced dyskinesia in the hemi-parkinsonian rat: role for interleukin-1beta. Neuroscience 156, 30–41. 10.1016/j.neuroscience.2008.07.016 18687386 PMC2615135

[B8] Beattie E. C. Stellwagen D. Morishita W. Bresnahan J. C. Ha B. K. Von Zastrow M.. (2002). Control of synaptic strength by glial TNFα. Science 295, 2282–2285. 10.1126/science.1067859 11910117

[B9] Bezard E. (2013). Experimental reappraisal of continuous dopaminergic stimulation against L-dopa-induced dyskinesia. Mov. Disord. 28, 1021–1022. 10.1002/mds.25251 23143999

[B10] Bezard E. Ferry S. Mach U. Stark H. Leriche L. Boraud T.. (2003). Attenuation of levodopa-induced dyskinesia by normalizing dopamine D3 receptor function. Nat. Med. 9, 762–767. 10.1038/nm875 12740572

[B11] Blanchet P. J. Gomez-Mancilla B. Di Paolo T. Bedard P. J. (1995). Is striatal dopaminergic receptor imbalance responsible for levodopa-induced dyskinesia? Fundam. Clin. Pharmacol. 9, 434–442. 10.1111/j.1472-8206.1995.tb00518.x 8617407

[B12] Blanchet P. J. Konitsiotis S. Chase T. N. (1998). Amantadine reduces levodopa-induced dyskinesias in parkinsonian monkeys. Mov. Disord. 13, 798–802. 10.1002/mds.870130507 9756148

[B13] Bortolanza M. Padovan-Neto F. E. Cavalcanti-Kiwiatkoski R. Dos Santos-Pereira M. Mitkovski M. Raisman-Vozari R.. (2015). Are cyclooxygenase-2 and nitric oxide involved in the dyskinesia of Parkinson’s disease induced by L-DOPA? Philos. Trans. R. Soc. Lond. B Biol. Sci. 370:20140190. 10.1098/rstb.2014.0190 26009769 PMC4455759

[B14] Braak H. Del Tredici K. Rub U. de Vos R. A. Jansen Steur E. N. Braak E. (2003). Staging of brain pathology related to sporadic Parkinson’s disease. Neurobiol. Aging 24, 197–211. 10.1016/s0197-4580(02)00065-9 12498954

[B15] Brodsky M. A. Hogarth P. Nutt J. G. (2006). OFF-off rebound dyskinesia in subthalamic nucleus deep brain stimulation of Parkinson’s disease. Mov. Disord. 21, 1487–1490. 10.1002/mds.20964 16721730

[B16] Calabresi P. Picconi B. Tozzi A. Di Filippo M. (2007). Dopamine-mediated regulation of corticostriatal synaptic plasticity. Trends Neurosci. 30, 211–219. 10.1016/j.tins.2007.03.001 17367873

[B17] Carlsson T. Carta M. Winkler C. Bjorklund A. Kirik D. (2007). Serotonin neuron transplants exacerbate L-DOPA-induced dyskinesias in a rat model of Parkinson’s disease. J. Neurosci. 27, 8011–8022. 10.1523/jneurosci.2079-07.2007 17652591 PMC6672736

[B19] Carta M. Carlsson T. Kirik D. Björklund A. (2007). Dopamine released from 5-HT terminals is the cause of L-DOPA-induced dyskinesia in parkinsonian rats. Brain 130, 1819–1833. 10.1093/brain/awm082 17452372

[B18] Carta A. R. Pinna A. Morelli M. (2006). How reliable is the behavioural evaluation of dyskinesia in animal models of Parkinson’s disease? Behav. Pharmacol. 17, 393–402. 10.1097/00008877-200609000-00005 16940760

[B23] Cenci M. A. Campbell K. Björklund A. (1993). Neuropeptide messenger RNA expression in the 6-hydroxydopamine-lesioned rat striatum reinnervated by fetal dopaminergic transplants: differential effects of the grafts on preproenkephalin, preprotachykinin and prodynorphin messenger RNA levels. Neuroscience 57, 275–296. 10.1016/0306-4522(93)90062-k 8115038

[B24] Cenci M. A. Kalén P. Mandel R. J. Wictorin K. Björklund A. (1992). Dopaminergic transplants normalize amphetamine- and apomorphine-induced Fos expression in the 6-hydroxydopamine-lesioned striatum. Neuroscience 46, 943–957. 10.1016/0306-4522(92)90196-9 1347413

[B20] Cenci M. A. Konradi C. (2010). Maladaptive striatal plasticity in L-DOPA-induced dyskinesia. Prog. Brain Res. 183, 209–233. 10.1016/s0079-6123(10)83011-0 20696322 PMC2930606

[B22] Cenci M. A. Lee C. S. Björklund A. (1998). L-DOPA-induced dyskinesia in the rat is associated with striatal overexpression of prodynorphin- and glutamic acid decarboxylase mRNA. Eur. J. Neurosci. 10, 2694–2706. 10.1046/j.1460-9568.1998.00285.x 9767399

[B21] Cenci M. A. Lundblad M. (2006). Post- versus presynaptic plasticity in L-DOPA-induced dyskinesia. J. Neurochem. 99, 381–392. 10.1111/j.1471-4159.2006.04124.x 16942598

[B25] Crittenden J. R. Graybiel A. M. (2011). Basal Ganglia disorders associated with imbalances in the striatal striosome and matrix compartments. Front. Neuroanat. 5:59. 10.3389/fnana.2011.00059 21941467 PMC3171104

[B26] Cubo E. Gracies J. M. Benabou R. Olanow C. W. Raman R. Leurgans S.. (2001). Early morning off-medication dyskinesias, dystonia and choreic subtypes. Arch. Neurol. 58, 1379–1382. 10.1001/archneur.58.9.1379 11559308

[B27] Di Loreto S. Florio T. Capozzo A. Napolitano A. Adorno D. Scarnati E. (1996). Transplantation of mesencephalic cell suspension in dopamine-denervated striatum of the rat. Exp. Neurol. 138, 318–326. 10.1006/exnr.1996.0070 8812168

[B280] Doucet G. Murata Y. Brundin P. Bosler O. Mons N. Geffard M. (1989). Host afferents into intrastriatal transplants of fetal ventral mesencephalon. Exp. Neurol. 106, 1–19. 10.1016/0014-4886(89)90139-8 2477271

[B28] Doucet J. P. Nakabeppu Y. Bedard P. J. Hope B. T. Nestler E. J. Jasmin B. J.. (1996). Chronic alterations in dopaminergic neurotransmission produce a persistent elevation of deltaFosB-like protein(s) in both the rodent and primate striatum. Eur. J. Neurosci. 8, 365–381. 10.1111/j.1460-9568.1996.tb01220.x 8714707

[B29] Encarnacion E. V. Hauser R. A. (2008). Levodopa-induced dyskinesias in Parkinson’s disease: etiology, impact on quality of life and treatments. Eur. Neurol. 60, 57–66. 10.1159/000131893 18480609

[B30] Fieblinger T. Graves S. M. Sebel L. E. Alcacer C. Plotkin J. L. Gertler T. S.. (2014). Cell type-specific plasticity of striatal projection neurons in parkinsonism and L-DOPA-induced dyskinesia. Nat. Commun. 5:5316. 10.1038/ncomms6316 25360704 PMC4431763

[B31] Fox S. H. Lang A. E. Brotchie J. M. (2006). Translation of nondopaminergic treatments for levodopa-induced dyskinesia from MPTP-lesioned nonhuman primates to phase IIa clinical studies: keys to success and roads to failure. Mov. Disord. 21, 1578–1594. 10.1002/mds.20936 16874752

[B32] Freed C. R. Greene P. E. Breeze R. E. Tsai W. Y. DuMouchel W. Kao R.. (2001). Transplantation of embryonic dopamine neurons for severe Parkinson’s disease. N. Engl. J. Med. 344, 710–719. 10.1056/nejm200103083441002 11236774

[B33] Freund T. F. Bolam J. P. Bjorklund A. Stenevi U. Dunnett S. B. Powell J. F.. (1985). Efferent synaptic connections of grafted dopaminergic neurons reinnervating the host neostriatum: a tyrosine hydroxylase immunocytochemical study. J. Neurosci. 5, 603–616. 2857778 10.1523/JNEUROSCI.05-03-00603.1985 PMC6565037

[B34] García J. Carlsson T. Döbrossy M. Nikkhah G. Winkler C. (2011a). Extent of pre-operative L-DOPA-induced dyskinesia predicts the severity of graft-induced dyskinesia after fetal dopamine cell transplantation. Exp. Neurol. 232, 270–279. 10.1016/j.expneurol.2011.09.017 21946270

[B35] García J. Carlsson T. Döbrössy M. Nikkhah G. Winkler C. (2011b). Impact of dopamine versus serotonin cell transplantation for the development of graft-induced dyskinesia in a rat Parkinsonian model. Neurobiol. Dis. 43, 576–587. 10.1016/j.brainres.2012.06.029 21600983

[B36] Gardoni F. Picconi B. Ghiglieri V. Polli F. Bagetta V. Bernardi G.. (2006). A critical interaction between NR2B and MAGUK in L-DOPA induced dyskinesia. J. Neurosci. 26, 2914–2922. 10.1523/jneurosci.5326-05.2006 16540568 PMC6673976

[B37] Gardoni F. Sgobio C. Pendolino V. Calabresi P. Di Luca M. Picconi B. (2012). Targeting NR2A-containing NMDA receptors reduces L-DOPA-induced dyskinesias. Neurobiol. Aging 33, 2138–2144. 10.1016/j.neurobiolaging.2011.06.019 21821315

[B38] Gerfen C. R. (1992). The neostriatal mosaic: multiple levels of compartmental organization. Trends Neurosci. 15, 133–139. 10.1016/0166-2236(92)90355-C 1374971

[B39] Grealish S. Diguet E. Kirkeby A. Mattsson B. Heuer A. Bramoulle Y.. (2014). Human ESC-derived dopamine neurons show similar preclinical efficacy and potency to fetal neurons when grafted in a rat model of Parkinson’s disease. Cell Stem Cell 15, 653–665. 10.1016/j.stem.2014.09.017 25517469 PMC4232736

[B40] Hagell P. Cenci M. A. (2005). Dyskinesias and dopamine cell replacement in Parkinson’s disease: a clinical perspective. Brain Res. Bull. 68, 4–15. 10.1016/j.brainresbull.2004.10.013 16324999

[B41] Hagell P. Piccini P. Björklund A. Brundin P. Rehncrona S. Widner H.. (2002). Dyskinesias following neural transplantation in Parkinson’s disease. Nat. Neurosci. 5, 627–628. 12042822 10.1038/nn863

[B42] Hely M. A. Morris J. G. Reid W. G. Trafficante R. (2005). Sydney multicenter study of Parkinson’s disease: non-L-dopa-responsive problems dominate at 15 years. Mov. Disord. 20, 190–199. 10.1002/mds.20324 15551331

[B43] Hwang W. J. Calne D. B. Tsui J. K. de la Fuente-Fernández R. (2001). The long-term response to levodopa in dopa-responsive dystonia. Parkinsonism Relat. Disord. 8, 1–5. 10.1016/s1353-8020(00)00084-5 11472874

[B44] Ingham C. A. Hood S. H. Taggart P. Arbuthnott G. W. (1998). Plasticity of synapses in the rat neostriatum after unilateral lesion of the nigrostriatal dopaminergic pathway. J. Neurosci. 18, 4732–4743. 9614247 10.1523/JNEUROSCI.18-12-04732.1998 PMC6792704

[B45] Juri C. Viviani P. Chaná P. (2008). Features associated with the development of non-motor manifestations in Parkinson’s disease. Arq. Neuropsiquiatr. 66, 22–25. 10.1590/s0004-282x2008000100006 18392408

[B46] Khlebtovsky A. Rigbi A. Melamed E. Ziv I. Steiner I. Gad A.. (2012). Patient and caregiver perceptions of the social impact of advanced Parkinson’s disease and dyskinesias. J. Neural Transm. (Vienna) 119, 1367–1371. 10.1007/s00702-012-0796-9 22437202

[B47] Kobylecki C. Crossman A. R. Ravenscroft P. (2013). Alternative splicing of AMPA receptor subunits in the 6-OHDA-lesioned rat model of Parkinson’s disease and L-DOPA-induced dyskinesia. Exp. Neurol. 247, 476–484. 10.1016/j.expneurol.2013.01.019 23360800

[B48] Konradi C. Westin J. E. Carta M. Eaton M. E. Kuter K. Dekundy A.. (2004). Transcriptome analysis in a rat model of L-DOPA-induced dyskinesia. Neurobiol. Dis. 17, 219–236. 10.1016/j.nbd.2004.07.005 15474360 PMC4208672

[B49] Kriks S. Shim J. W. Piao J. Ganat Y. M. Wakeman D. R. Xie Z.. (2011). Dopamine neurons derived from human ES cells efficiently engraft in animal models of Parkinson’s disease. Nature 480, 547–551. 10.1038/nature10648 22056989 PMC3245796

[B50] Lane E. L. (2011). Clinical and experimental experiences of graft-induced dyskinesia. Int. Rev. Neurobiol. 98, 173–186. 10.1016/b978-0-12-381328-2.00007-9 21907087

[B51] Lane E. L. Björklund A. Dunnett S. Winkler C. (2010). Neural grafting in Parkinson’s disease: unraveling the mechanisms underlying graft-induced dyksinesia. Prog. Brain Res. 184, 295–309. 10.1016/S0079-6123(10)84015-4 20887881

[B53] Lane E. L. Soulet D. Vercammen L. Cenci M. A. Brundin P. (2008). Neuroinflammation in the generation of post-transplantation dyskinesia in Parkinson’s disease. Neurobiol. Dis. 32, 220–228. 10.1016/j.nbd.2008.06.011 18675359

[B54] Lane E. L. Vercammen L. Cenci M. A. Brundin P. (2009). Priming for L-DOPA-induced abnormal involuntary movements increases the severity of amphetamine-induced dyskinesia in grafted rats. Exp. Neurol. 219, 355–358. 10.1016/j.expneurol.2009.04.010 19393238

[B52] Lane E. L. Winkler C. Brundin P. Cenci M. A. (2006). The impact of graft size on the development of dyskinesia following intrastriatal grafting of embryonic dopamine neurons in the rat. Neurobiol. Dis. 22, 334–345. 10.1016/j.nbd.2005.11.011 16406222

[B55] Leranth C. Sladek J. R. Jr. Roth R. H. Redmond D. E. Jr. (1998). Efferent synaptic connections of dopaminergic neurons grafted into the caudate nucleus of experimentally induced parkinsonian monkeys are different from those of control animals. Exp. Brain Res. 123, 323–333. 10.1007/s002210050575 9860271

[B56] Lindgren H. S. Andersson D. R. Lagerkvist S. Nissbrandt H. Cenci M. A. (2010). L-DOPA-induced dopamine efflux in the striatum and the substantia nigra in a rat model of Parkinson’s disease: temporal and quantitative relationship to the expression of dyskinesia. J. Neurochem. 112, 1465–1476. 10.1111/j.1471-4159.2009.06556.x 20050978

[B57] Lindgren H. S. Rylander D. Iderberg H. Andersson M. O’Sullivan S. S. Williams D. R.. (2011). Putaminal upregulation of FosB/ΔFosB-like immunoreactivity in Parkinson’s disease patients with dyskinesia. J. Parkinsons Dis. 1, 347–357. 10.3233/JPD-2011-11068 23933656

[B58] Lundblad M. Andersson M. Winkler C. Kirik D. Wierup N. Cenci M. A. (2002). Pharmacological validation of behavioural measures of akinesia and dyskinesia in a rat model of Parkinson’s disease. Eur. J. Neurosci. 15, 120–132. 10.1046/j.0953-816x.2001.01843.x 11860512

[B59] Ma Y. Feigin A. Dhawan V. Fukuda M. Shi Q. Greene P.. (2002). Dyskinesia after fetal cell transplantation for Parkinsonism: a PET study. Ann. Neurol. 52, 628–634. 10.1002/ana.10359 12402261

[B60] Maeda T. Nagata K. Yoshida Y. Kannari K. (2005). Serotonergic hyperinnervation into the dopaminergic denervated striatum compensates for dopamine conversion from exogenously administered l-DOPA. Brain Res. 1046, 230–233. 10.1016/j.brainres.2005.04.019 15894297

[B61] Mahalik T. J. Clayton G. H. (1991). Specific outgrowth from neurons of ventral mesencephalic grafts to the catecholamine-depleted striatum of adult hosts. Exp. Neurol. 113, 18–27. 10.1016/0014-4886(91)90141-x 1710572

[B62] Mahalik T. J. Finger T. E. Stromberg I. Olson L. (1985). Substantia nigra transplants into denervated striatum of the rat: ultrastructure of graft and host interconnections. J. Comp. Neurol. 240, 60–70. 10.1002/cne.902400105 2865279

[B63] Manson A. Stirpe P. Schrag A. (2012). Levodopa-induced-dyskinesias clinical features, incidence, risk factors, management and impact on quality of life. J. Parkinsons Dis. 2, 189–198. 10.3233/JPD-2012-120103 23938226

[B64] Maries E. Kordower J. H. Chu Y. Collier T. J. Sortwell C. E. Olaru E.. (2006). Focal not widespread grafts induce novel dyskinetic behavior in parkinsonian rats. Neurobiol. Dis. 21, 165–180. 10.1016/j.nbd.2005.07.002 16095907

[B65] Marrone D. F. Petit T. L. (2002). The role of synaptic morphology in neural plasticity: structural interactions underlying synaptic power. Brain Res. Brain Res. Rev. 38, 291–308. 10.1016/s0165-0173(01)00147-3 11890978

[B66] Mellone M. Stanic J. Hernandez L. F. Iglesias E. Zianni E. Longhi A.. (2015). NMDA receptor GluN2A/GluN2B subunit ratio as synaptic trait of levodopa-induced dyskinesias: from experimental models to patients. Front. Cell. Neurosci. 9:245. 10.3389/fncel.2015.00245 26217176 PMC4491616

[B67] Mendez I. Sanchez-Pernaute R. Cooper O. Viñuela A. Ferrari D. Björklund L.. (2005). Cell type analysis of functional fetal dopamine cell suspension transplants in the striatum and substantia nigra of patients with Parkinson’s disease. Brain 128, 1498–1510. 10.1093/brain/awh510 15872020 PMC2610438

[B68] Merello M. Perez-Lloret S. Antico J. Obeso J. A. (2006). Dyskinesias induced by subthalamotomy in Parkinson’s disease are unresponsive to amantadine. J. Neurol. Neurosurg. Psychiatry 77, 172–174. 10.1136/jnnp.2005.068940 16421117 PMC2077561

[B69] Mones R. J. (1970). Parkinson’s disease. I. N Y State J. Med. 70, 2687–2691. 4919227

[B70] Montel S. Bonnet A. M. Bungener C. (2009). Quality of life in relation to mood, coping strategies and dyskinesia in Parkinson’s disease. J. Geriatr. Psychiatry Neurol. 22, 95–102. 10.1177/0891988708328219 19150974

[B71] Morgante F. Espay A. J. Gunraj C. Lang A. E. Chen R. (2006). Motor cortex plasticity in Parkinson’s disease and levodopa-induced dyskinesias. Brain 129(Pt. 4), 1059–1069. 10.1093/brain/awl031 16476674

[B72] Muñoz A. Lopez-Real A. Labandeira-Garcia J. L. Guerra M. J. (2003). Interaction between the noradrenergic and serotonergic systems in locomotor hyperactivity and striatal expression of Fos induced by amphetamine in rats. Exp. Brain Res. 153, 92–99. 10.1007/s00221-003-1582-6 12955385

[B73] Nutt J. G. (1990). Levodopa-induced dyskinesia: review, observations and speculations. Neurology 40, 340–345. 10.1212/wnl.40.2.340 2405297

[B74] Olanow C. W. Goetz C. G. Kordower J. H. Stoessl A. J. Sossi V. Brin M. F.. (2003). A double-blind controlled trial of bilateral fetal nigral transplantation in Parkinson’s disease. Ann. Neurol. 54, 403–414. 10.1002/ana.10720 12953276

[B75] Olanow C. W. Gracies J. M. Goetz C. G. Stoessl A. J. Freeman T. Kordower J. H.. (2009). Clinical pattern and risk factors for dyskinesias following fetal nigral transplantation in Parkinson’s disease: a double blind video-based analysis. Mov. Disord. 24, 336–343. 10.1002/mds.22208 19006186

[B76] Ossola B. Schendzielorz N. Chen S. H. Bird G. S. Tuominen R. K. Mannistö P. T.. (2011). Amantadine protects dopamine neurons by a dual action: reducing activation of microglia and inducing expression of GDNF in astroglia [corrected]. Neuropharmacology 61, 574–582. 10.1016/j.neuropharm.2011.04.030 21586298 PMC3130082

[B77] Paillé V. Picconi B. Bagetta V. Ghiglieri V. Sgobio C. Di Filippo M.. (2010). Distinct levels of dopamine denervation differentially alter striatal synaptic plasticity and NMDA receptor subunit composition. J. Neurosci. 30, 14182–14193. 10.1523/JNEUROSCI.2149-10.2010 20962239 PMC6634757

[B78] Pavón N. Martin A. B. Mendialdua A. Moratalla R. (2006). ERK phosphorylation and FosB expression are associated with L-DOPA-induced dyskinesia in hemiparkinsonian mice. Biol. Psychiatry 59, 64–74. 10.1016/j.biopsych.2005.05.044 16139809

[B79] Peters A. Palay S. L. (1991). The morphology of synapses. J. Neurocytol. 25, 687–700. 10.1007/BF02284835 9023718

[B81] Piccini P. Lindvall O. Björklund A. Brundin P. Hagell P. Ceravolo R.. (2000). Delayed recovery of movement-related cortical function in Parkinson’s disease after striatal dopaminergic grafts. Ann. Neurol. 48, 689–695. 10.1002/1531-8249(200011)48:5<689::aid-ana1>3.3.co;2-e 11079531

[B80] Piccini P. Pavese N. Hagell P. Reimer J. Björklund A. Oertel W. H.. (2005). Factors affecting the clinical outcome after neural transplantation in Parkinson’s disease. Brain 128, 2977–2986. 10.1093/brain/awh649 16246865

[B83] Picconi B. Bagetta V. Ghiglieri V. Paillè V. Di Filippo M. Pendolino V.. (2011). Inhibition of phosphodiesterases rescues striatal long-term depression and reduces levodopa-induced dyskinesia. Brain 134, 375–387. 10.1093/brain/awq342 21183486

[B82] Picconi B. Centonze D. Hakansson K. Bernardi G. Greengard P. Fisone G.. (2003). Loss of bidirectional striatal synaptic plasticity in L-DOPA-induced dyskinesia. Nat. Neurosci. 6, 501–506. 10.1038/nn1040 12665799

[B84] Pietracupa S. Fasano A. Fabbrini G. Sarchioto M. Bloise M. Latorre A.. (2013). Poor self-awareness of levodopa-induced dyskinesias in Parkinson’s disease: clinical features and mechanisms. Parkinsonism Relat. Disord. 19, 1004–1008. 10.1016/j.parkreldis.2013.07.002 23890762

[B85] Politis M. Loane C. (2011). Serotonergic dysfunction in Parkinson’s disease and its relevance to disability. ScientificWorldJournal 11, 1726–1734. 10.1100/2011/172893 22125431 PMC3201695

[B86] Politis M. Wu K. Loane C. Brooks D. J. Kiferle L. Turkheimer F. E.. (2014). Serotonergic mechanisms responsible for levodopa-induced dyskinesias in Parkinson’s disease patients. J. Clin. Invest. 124, 1340–1349. 10.1172/jci71640 24531549 PMC3934188

[B87] Politis M. Wu K. Loane C. Quinn N. P. Brooks D. J. Rehncrona S.. (2010). Serotonergic neurons mediate dyskinesia side effects in Parkinson’s patients with neural transplants. Sci. Transl. Med. 2:38ra46. 10.1126/scitranslmed.3000976 20592420

[B88] Pons R. Syrengelas D. Youroukos S. Orfanou I. Dinopoulos A. Cormand B.. (2013). Levodopa-induced dyskinesias in tyrosine hydroxylase deficiency. Mov. Disord. 28, 1058–1063. 10.1002/mds.25382 23389938

[B89] Radja F. Descarries L. Dewar K. M. Reader T. A. (1993). Serotonin 5-HT1 and 5-HT2 receptors in adult rat brain after neonatal destruction of nigrostriatal dopamine neurons: a quantitative autoradiographic study. Brain Res. 606, 273–285. 10.1016/0006-8993(93)90995-y 8490720

[B90] Rascol O. (2000). The pharmacological therapeutic management of levodopa-induced dyskinesias in patients with Parkinson’s disease. J. Neurol. 247, II51–II57. 10.1007/pl00007761 10991666

[B91] Redmond D. E. Jr. Vinuela A. Kordower J. H. Isacson O. (2008). Influence of cell preparation and target location on the behavioral recovery after striatal transplantation of fetal dopaminergic neurons in a primate model of Parkinson’s disease. Neurobiol. Dis. 29, 103–116. 10.1016/j.nbd.2007.08.008 17920901 PMC2174366

[B92] Rumpel R. Alam M. Klein A. Ozer M. Wesemann M. Jin X.. (2013). Neuronal firing activity and gene expression changes in the subthalamic nucleus after transplantation of dopamine neurons in hemiparkinsonian rats. Neurobiol. Dis. 59, 230–243. 10.1016/j.nbd.2013.07.016 23938762

[B93] Rylander D. (2013). Restoration of synaptic plasticity in the host striatum: can transplants make it? Neuroreport 24, 1016–1018. 10.1097/wnr.0000000000000061 24152765

[B95] Rylander D. Bagetta V. Pendolino V. Zianni E. Grealish S. Gardoni F.. (2013). Region-specific restoration of striatal synaptic plasticity by dopamine grafts in experimental parkinsonism. Proc. Natl. Acad. Sci. U S A 110, E4375–E4384. 10.1073/pnas.1311187110 24170862 PMC3831970

[B94] Rylander D. Parent M. O’Sullivan S. S. Dovero S. Lees A. J. Bezard E.. (2010). Maladaptive plasticity of serotonin axon terminals in levodopa-induced dyskinesia. Ann. Neurol. 68, 619–628. 10.1002/ana.22097 20882603

[B96] Santini E. Valjent E. Usiello A. Carta M. Borgkvist A. Girault J. A.. (2007). Critical involvement of cAMP/DARPP-32 and extracellular signal-regulated protein kinase signaling in L-DOPA-induced dyskinesia. J. Neurosci. 27, 6995–7005. 10.1523/jneurosci.0852-07.2007 17596448 PMC6672217

[B97] Schuster S. Doudnikoff E. Rylander D. Berthet A. Aubert I. Ittrich C.. (2009). Antagonizing L-type Ca2+ channel reduces development of abnormal involuntary movement in the rat model of L-3,4-dihydroxyphenylalanine-induced dyskinesia. Biol. Psychiatry 65, 518–526. 10.1016/j.biopsych.2008.09.008 18947822

[B98] Shen W. Plotkin J. L. Francardo V. Ko W. K. Xie Z. Li Q.. (2015). M4 muscarinic receptor signaling ameliorates striatal plasticity deficits in models of L-DOPA-induced dyskinesia. Neuron 88, 762–773. 10.1016/j.neuron.2015.10.039 26590347 PMC4864040

[B99] Shin E. García J. Winkler C. Bjorklund A. Carta M. (2012). Serotonergic and dopaminergic mechanisms in graft-induced dyskinesia in a rat model of Parkinson’s disease. Neurobiol. Dis. 47, 393–406. 10.1016/j.nbd.2012.03.038 22579773

[B100] Simmons D. A. Belichenko N. P. Yang T. Condon C. Monbureau M. Shamloo M.. (2013). A small molecule TrkB ligand reduces motor impairment and neuropathology in R6/2 and BACHD mouse models of Huntington’s disease. J. Neurosci. 33, 18712–18727. 10.1523/JNEUROSCI.1310-13.2013 24285878 PMC3841443

[B102] Smith G. A. Breger L. S. Lane E. L. Dunnett S. B. (2012). Pharmacological modulation of amphetamine-induced dyskinesia in transplanted hemi-parkinsonian rats. Neuropharmacology 63, 818–828. 10.1016/j.neuropharm.2012.06.011 22722025

[B101] Smith G. A. Murphy E. Dunnett S. Lane E. L. (2011). “Induced animal models of Parkinson’s disease,” in Handbook of Laboratory Animal Science, Animal Models (Vol. 2), eds Hau J. Schapiro S. J. (Boca Raton, FL: Taylor and Francis group, LLC), 75–96.

[B103] Soderstrom K. E. Meredith G. Freeman T. B. McGuire S. O. Collier T. J. Sortwell C. E.. (2008). The synaptic impact of the host immune response in a parkinsonian allograft rat model: influence on graft-derived aberrant behaviors. Neurobiol. Dis. 32, 229–242. 10.1016/j.nbd.2008.06.018 18672063 PMC2886670

[B104] Soderstrom K. E. O’Malley J. A. Levine N. D. Sortwell C. E. Collier T. J. Steece-Collier K.. (2010). Impact of dendritic spine preservation in medium spiny neurons on dopamine graft efficacy and the expression of dyskinesias in parkinsonian rats. Eur. J. Neurosci. 31, 478–490. 10.1111/j.1460-9568.2010.07077.x 20105237 PMC2940228

[B105] Solís O. Garcia-Montes J. R. González-Granillo A. Xu M. Moratalla R. (2015). Dopamine D3 receptor modulates l-DOPA-induced dyskinesia by targeting D1 receptor-mediated striatal signaling. Cereb. Cortex [Epub ahead of print]. 10.1093/cercor/bhv231 26483399 PMC5939228

[B106] Spillantini M. G. Schmidt M. L. Lee V. M. Trojanowski J. Q. Jakes R. Goedert M. (1997). Alpha-synuclein in lewy bodies. Nature 388, 839–840. 10.1038/42166 9278044

[B107] Steece-Collier K. Soderstrom K. E. Collier T. J. Sortwell C. E. Maries-Lad E. (2009). Effect of levodopa priming on dopamine neuron transplant efficacy and induction of abnormal involuntary movements in parkinsonian rats. J. Comp. Neurol. 515, 15–30. 10.1002/cne.22037 19399877 PMC2886671

[B108] Stromberg I. Kehr J. Fuxe K. (2000). Restoration of dopamine transmission in graft reinnervated striatum. Evidence for regulation of dopamine D2 receptor function in regions lacking dopamine. Prog. Brain Res. 125, 309–315. 10.1016/s0079-6123(00)25020-6 11098667

[B109] Suárez L. M. Solís O. Caramés J. M. Taravini I. R. Solís J. M. Murer M. G.. (2014). L-DOPA treatment selectively restores spine density in dopamine receptor D2-expressing projection neurons in dyskinetic mice. Biol. Psychiatry 75, 711–722. 10.1016/j.biopsych.2013.05.006 23769604

[B110] Takahashi H. Ohama E. Suzuki S. Horikawa Y. Ishikawa A. Morita T.. (1994). Familial juvenile parkinsonism: clinical and pathologic study in a family. Neurology 44, 437–441. 10.1212/wnl.44.3_part_1.437 8145912

[B111] Tanaka H. Kannari K. Maeda T. Tomiyama M. Suda T. Matsunaga M. (1999). Role of serotonergic neurons in L-DOPA-derived extracellular dopamine in the striatum of 6-OHDA-lesioned rats. Neuroreport 10, 631–634. 10.1097/00001756-199902250-00034 10208602

[B112] Tønnesen J. Parish C. L. Sorensen A. T. Andersson A. Lundberg C. Deisseroth K.. (2011). Functional integration of grafted neural stem cell-derived dopaminergic neurons monitored by optogenetics in an *in vitro* Parkinson model. PLoS One 6:e17560. 10.1371/journal.pone.0017560 21394212 PMC3048875

[B113] Torper O. Ottosson D. R. Pereira M. Lau S. Cardoso T. Grealish S.. (2015). *In Vivo* reprogramming of striatal NG2 glia into functional neurons that integrate into local host circuitry. Cell Rep. 12, 474–481. 10.1016/j.celrep.2015.06.040 26166567 PMC4521079

[B118] Vinuela A. Hallett P. J. Reske-Nielsen C. Patterson M. Sotnikova T. D. Caron M. G.. (2008). Implanted reuptake-deficient or wild-type dopaminergic neurons improve ON L-dopa dyskinesias without OFF-dyskinesias in a rat model of Parkinson’s disease. Brain 131(Pt. 12), 3361–3379. 10.1093/brain/awn192 18988638 PMC2639209

[B114] Wang Q. Liu Y. Zhou J. (2015). Neuroinflammation in Parkinson’s disease and its potential as therapeutic target. Transl. Neurodegener. 4:19. 10.1186/s40035-015-0042-0 26464797 PMC4603346

[B115] Weintraub D. Koester J. Potenza M. N. Siderowf A. D. Stacy M. Voon V.. (2010). Impulse control disorders in Parkinson disease: a cross-sectional study of 3090 patients. Arch. Neurol. 67, 589–595. 10.1001/archneurol.2010.65 20457959

[B116] Westin J. E. Vercammen L. Strome E. M. Konradi C. Cenci M. A. (2007). Spatiotemporal pattern of striatal ERK1/2 phosphorylation in a rat model of L-DOPA-induced dyskinesia and the role of dopamine D1 receptors. Biol. Psychiatry 62, 800–810. 10.1016/j.biopsych.2006.11.032 17662258 PMC4205578

[B117] Winkler C. Bentlage C. Nikkhah G. Samii M. Björklund A. (1999). Intranigral transplants of GABA-rich striatal tissue induce behavioral recovery in the rat Parkinson model and promote the effects obtained by intrastriatal dopaminergic transplants. Exp. Neurol. 155, 165–186. 10.1006/exnr.1998.6916 10072293

[B119] Yukhananov R. Kissin I. (2008). Persistent changes in spinal cord gene expression after recovery from inflammatory hyperalgesia: a preliminary study on pain memory. BMC Neurosci. 9:32. 10.1186/1471-2202-9-32 18366630 PMC2315656

[B120] Zhang Y. Meredith G. E. Mendoza-Elias N. Rademacher D. J. Tseng K. Y. Steece-Collier K.. (2013). Aberrant restoration of spines and their synapses in L-DOPA-induced dyskinesia: involvement of corticostriatal but not thalamostriatal synapses. J. Neurosci. 33, 11655–11667. 10.1523/JNEUROSCI.0288-13.2013 23843533 PMC3724545

